# Prognostic Factors of Pediatric Acute Myeloid Leukemia Patients with t(8;21) (q22;q22): A Single-Center Retrospective Study

**DOI:** 10.3390/children11050605

**Published:** 2024-05-17

**Authors:** Jiapeng Yang, Xiaohua Zhu, Honghong Zhang, Yang Fu, Zifeng Li, Ziping Xing, Yi Yu, Ping Cao, Jun Le, Junye Jiang, Jun Li, Hongsheng Wang, Xiaowen Zhai

**Affiliations:** Department of Hematology and Oncology, National Children’s Medical Center, Children’s Hospital of Fudan University, Shanghai 201102, China

**Keywords:** t(8;21), pediatric acute myeloid leukemia, prognosis, loss of sex chromosome, extramedullary leukemia

## Abstract

This retrospective study aimed to analyze the treatment effect and prognostic factors of pediatric acute myeloid leukemia (AML) patients with t(8;21). A total of 268 newly diagnosed pediatric AML (pAML) enrolled from 1 January 2005 to 31 December 2022 were retrospectively reviewed, and 50 (18.7%) patients harbored t(8;21) translocation. CR rate, OS, EFS, and RFS were assessed by multivariate Logistic and Cox regression models in these patients. Of the 50 patients, 2 patients abandoned treatment during the first induction course. Of the remaining 48 patients who received double-induction therapy and were included in the final analyses, CR1 and CR2 were 75.0% (36/48) and 95.8% (46/48), respectively. The overall three-year OS, EFS, and RFS were 68.4% (95% CI, 55.0–85.1), 64.2% (95% CI, 50.7–81.4), and 65.5% (95% CI, 51.9–82.8), respectively. The presence of loss of sex chromosome (LOS) at diagnosis (n = 21) was associated with a better 3-year OS [87.5% (95% CI, 72.7–100) vs. 52.7% (95% CI, 35.1–79.3), *p* = 0.0089], 3-year EFS [81.6% (95% CI, 64.7–100) vs. 49.7% (95% CI, 32.4–76.4), *p* = 0.023], and 3-year RFS [81.6% (95% CI, 64.7–100) vs. 51.7% (95% CI, 33.9–78.9), *p* = 0.036] than those without LOS (n = 27), and it was also an independent good prognostic factor of OS (HR, 0.08 [95% CI, 0.01–0.48], *p* = 0.005), EFS (HR, 0.22 [95% CI, 0.05–0.85], *p* = 0.029), and RFS (HR, 0.21 [95% CI, 0.05–0.90], *p* = 0.035). However, extramedullary leukemia (EML) featured the independent risk factors of inferior OS (HR, 10.99 [95% CI, 2.08–58.12], *p* = 0.005), EFS (HR, 4.75 [95% CI, 1.10–20.61], *p* = 0.037), and RFS (HR, 6.55 [95% CI, 1.40–30.63], *p* = 0.017) in pediatric individuals with t(8;21) AML. Further analysis of combining LOS with EML indicated that the EML+LOS− subgroup had significantly inferior OS (92.9%, [95% CI, 80.3–100]), EFS (86.2%, [95% CI, 70.0–100]), and RFS (86.2%, [95% CI, 80.3–100]) compared to the other three subgroups (all *p* < 0.001). LOS and EML are independent prognostic factors of OS, EFS, and RFS with t(8;21) pAML patients. LOS combined with EML may help improve risk stratification.

## 1. Introduction

Acute myeloid leukemia (AML) constitutes 20% of pediatric leukemias, with 5.1% of AML patients being diagnosed at an age younger than 20 years [1,2,3]. *AML1::ETO* (also known as *RUNX1::RUNX1T1*) resulting from t(8;21) (q22;q22) is one of the most frequent genetic aberrations, with a incidence of 20% in newly diagnosed pAML patients [4,5,6,7]. Although t(8;21) AML is generally categorized as a favorable subgroup, 20%–35% of patients with this genotype experience relapse, and the outcomes of this genotype are heterogeneous [8,9,10]. We conducted a single-center retrospective study of children with this genotype from 1 January 2005 to 31 December 2022 to search for prognostic factors that could potentially be useful to further improve outcomes and update therapy strategies.

## 2. Materials and Methods

### 2.1. Study Design and Patients

Individuals aged ≤eighteen years with a confirmed diagnosis of AML were enrolled in the retrospective study. Data from individuals without proof of t(8;21) presence and individuals without treatment during the first induction course were excluded from analyses. All patients were treated in the Children’s Hospital of Fudan University between 2005 and 2023, receiving risk-stratified therapy without prophylactic cranial irradiation. Patients treated in our institution from 2005 to 2014 received the CHFU-AML 2005 regimen, and CCLG-AML-2015 and CALSIII-AML18 regimens were used in 2015–2018 and 2018–2023, respectively [11,12]. The study was approved by the institutional review board of the Children’s Hospital of Fudan University, and informed consent was obtained from all patients or their legal guardians. Outcome data reported here were updated on 21 March 2023.

### 2.2. Morphology, Cytogenetics and Immunophenotyping

The morphologic assessment was founded on May-Grünwald–Giemsa stains, myeloperoxidase reactions, and nonspecific esterases utilizing α-naphthyl acetate following the FAB and WHO classifications [13,14,15]. A chromosome G-banding analysis was conducted on all cases according to standard procedures [16,17]. The description of karyotypes followed the ISCN [18]. Loss of sex chromosomes (LOS) was defined as the presence of a -X/-Y clone in more than 3 of the 20 cells analyzed [19]. Complex karyotype (CK) was defined as ≥3 chromosomal aberrations [20]. Monosomal karyotype (MK) was defined as ≥2 separate autosomal monosomies or 1 monosomy plus ≥ 1 structural aberrations [21,22]. Immunophenotyping was conducted in bone marrow (BM) specimens of all cases following previous descriptions [23,24]. Quantification of minimal residual disease (MRD) in the BM (after induction therapy I and II, or other later timepoint) using multiparameter flow cytometry (MFCM) commenced in June 2018.

### 2.3. Molecular Biology Analysis

Molecular studies were performed from June 2018, and the results for 20 patients are available. Mononuclear cells were isolated, and both DNA and mRNA were extracted from BM and peripheral blood samples. Additionally, random-primed cDNA synthesis was conducted according to the protocols [25]. As for fusion genes, interphase fluorescence in situ hybridization (FISH) with probes for RUNX1 and RUNX1T1 was conducted using commercially available probes. Investigations for *FLT3-ITD*, *C-KIT/D816*, *NPM1* (Exon12), and *CEBPA* were carried out in these patients [26].

### 2.4. Statistical Analysis

Each statistical assessment was performed utilizing R software (version 4.1.0). Outcomes were evaluated by using complete remission (CR), overall survival (OS), event-free survival (EFS), and relapse-free survival (RFS) (Table S1) [27]. Categorical variables were compared between groups utilizing the χ2 test and continuous variables by the Mann–Whitney U test. To identify factors associated with CR and prognosis, variables with *p* < 0.2 [28] in univariate analyses entered a multivariate Logistic regression model and a multivariate Cox regression model, respectively. Results were presented as OR or HR and 95% CI. Survival outcomes were determined utilizing the Kaplan–Meier method, and comparisons between groups were conducted utilizing the logrank test. Statistical importance was denoted by two-sided *p* < 0.05.

## 3. Results

### 3.1. Clinical Characteristics

**Figure 1:**
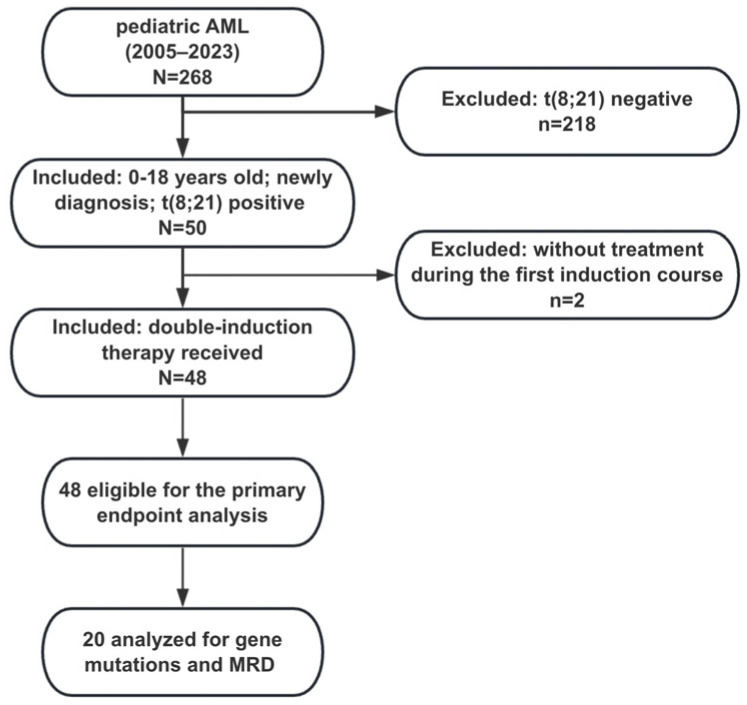
Flow diagram.

From 1 January 2005 to 31 December 2022, 268 evaluable individuals were enrolled from a Chinese children’s medical center. Data from 218 individuals without proven t(8;21) presence and two individuals without treatment during the first induction course were excluded (Figure 1). An amount of 48 individuals with t(8;21) AML were included in the final analyses, with a male-to-female ratio of 1.8:1. The median age at diagnosis was 8.6 years (range, 2.5–16.7), with 46 patients (95.8%) aged between 3 and 14 years and only one patient (2.1%) in each age group younger than three years and older than fourteen years, respectively. The median white blood cell (WBC) count was 15.9 × 10^9^/L (range, 1.7–123.0). The median platelet count was 28.5 × 10^9^/L (range, 4–265). The median hemoglobin (Hb) count was 78.6 g/L (38.2–122.0). The median follow-up time was 35.5 months (IQR, 12.8–74.8). According to MFCM, the sums of the percentage of positive cells for CD34 plus CD117 or HLA-DR were greater than 95% in these cases. CD19 antigen and CD56 antigen were detected in 23 (47.9%) and 21 (43.6%) of these cases, respectively. As for additional chromosome abnormality (ACA), t(8;21) was the sole cytogenetic aberration in 13 patients (27.1%), while 35 (72.9%) harbored other ACAs, 21 (43.8%) had loss of sex chromosome (LOS), 7 (14.6%) had CK, 11 (22.9%) had MK, 3 (6.3%) had deletion of chromosome 9q [del(9q)], only one (2.1%) had trisomy 4, and no one had trisomy 8. Of the 20 patients analyzed for genetic mutation, two (10.0%) were *FLT3-ITD* positive, 12 (60.0%) were *C-KIT* positive, and none of them had *CEBPA* and *NPM1* mutations. Within the same cohort evaluable for MRD analysis, 7 (35%) and 18 (90%) had a negative MRD status (defined by <0.1%) after induction therapy I and II, respectively (Table 1).

### 3.2. Treatment Outcome

**Figure 2:**
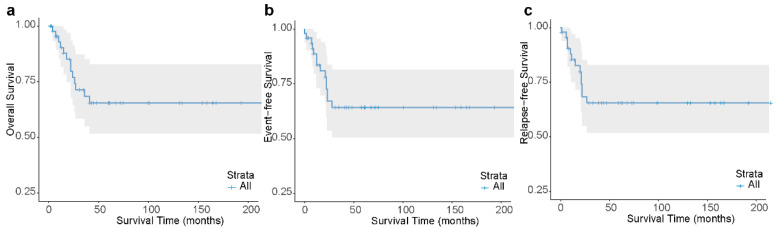
Outcomes of t(8;21) pAML patients. Kaplan–Meier analysis of overall survival (OS) (**a**), event-free survival (EFS) (**b**) and relapse-free survival (RFS) (**c**) for all patients. Three-year OS, EFS and RFS: 0.68 ± 0.08, 0.64 ± 0.08, 0.66 ± 0.08, respectively.

Overall, first complete remission (CR1) and second complete remission (CR2) were achieved by 75.0% (n = 36) and 95.8% (n = 46) of the patients, respectively. CR was achieved in 20 (100.0%) patients treated with the CALSIII-AML18 protocol, of whom 18 (90.0%) had a negative MRD status at the end of induction therapy. The 3-year OS, EFS, and RFS were 68.4% (95% CI, 55.0–85.1), 64.2% (95% CI, 50.7–81.4), and 65.5% (95% CI, 51.9–82.8), respectively (Figure 2a–c). The 3-year OS, EFS, and RFS of 18 cases treated with the CHFU-AML 2005 protocol were 72.2% (95% CI, 54.2–96.2), 61.1% (95% CI, 42.3–88.3), and 64.7% (95% CI, 45.5–91.9), respectively. The 3-year OS, EFS, and RFS of 10 cases treated with the CCLG-AML 2015 protocol were 60.0% (95% CI, 36.2–99.5), 60.0% (95% CI, 36.2–99.5), and 60.0% (95% CI, 36.2–99.5), respectively. The 3-year OS, EFS, and RFS of 20 cases treated with the CALSIII-AML18 protocol were 68.2% (95% CI, 43.8–100), 71.1% (95% CI, 47.5–100), and 71.1% (95% CI, 47.5–100), respectively. No significant differences were found in the outcomes (Figure S1) and CR rate among these protocols (Tables S2 and S3).

**Figure 3:**
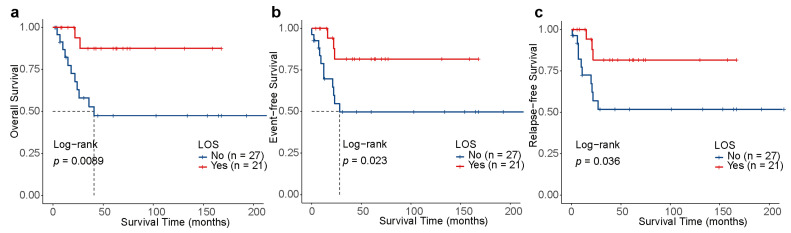
Outcomes depending on the loss of sex chromosomes (LOS) in t(8;21) AML. Kaplan–Meier analysis of OS (**a**), EFS (**b**) and RFS (**c**) for patients stratified by LOS.

**Figure 4:**
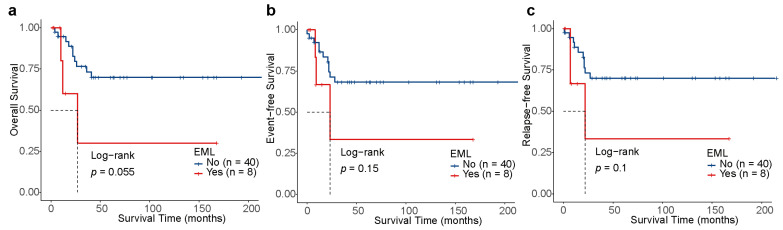
Outcomes depending on the extramedullary leukemia (EML) in t(8;21) AML. Kaplan–Meier analysis of OS (**a**), EFS (**b**) and RFS (**c**) for patients stratified by EML.

Of note, patients with LOS (n = 21) had significantly better 3-year OS [87.5% (95% CI, 72.7–100) vs. 52.7% (95% CI, 35.1–79.3), *p* = 0.0089], 3-year EFS [81.6% (95% CI, 64.7–100) vs. 49.7% (95% CI, 32.4–76.4), *p* = 0.023], and 3-year RFS [81.6% (95% CI, 64.7–100) vs. 51.7% (95% CI, 33.9–78.9), *p* = 0.036] than those without LOS (n = 27, Figure 3a–c). Additionally, the OS, EFS, and RFS of patients with EML did not have any significant differences to those without EML (*p* > 0.05, Figure 4a–c), which may be associated with the limited quantity of individuals with EML (n = 8). For the 20 patients treated under the CALSIII-AML18 regimen, there was no significant difference in prognosis between those with and without *C-KIT*/negative MRD status, respectively (all *p* > 0.05, Figures S2–S4).

### 3.3. Impact of Clinical and Biological Characteristics on CR Achievement

We assessed the association of clinical and biological features with CR achievement in a logistic regression model. As shown in Table 2, age at diagnosis, WBC, PB blast, FAB, CD19, MK, and Year of diagnosis were significantly linked to CR achievement of those patients. We then included these seven variables in the multivariate logistic analysis and revealed that none of these values remained significantly different for CR achievement.

### 3.4. Prognostic Factors for OS, EFS, and RFS

To assess the significant prognostic factors, Cox regression modeling was performed. As shown in Table 3 and Tables S4–S8, EML and LOS were significantly correlated with the OS, EFS, and RFS of t(8;21) pAML patients. Individuals with EML had lower OS (*p* = 0.071), EFS (*p* = 0.164), and RFS (*p* = 0.108), while those with LOS had higher OS (*p* = 0.022), EFS (*p* = 0.036), and RFS (*p* = 0.051). We then included the two covariates in the multivariate analyses and demonstrated that EML was an independent risk factor for inferior OS (hazard ratio [HR], 10.99 [95% CI, 2.08–58.12], *p* = 0.005), EFS (HR, 4.75 [95% CI, 1.10–20.61], *p* = 0.037), and RFS (HR, 6.55 [95% CI, 1.40–30.63], *p* = 0.017), while LOS was an independent good prognostic factor that influenced OS (HR, 0.08 [95% CI, 0.01–0.48], *p* = 0.005), EFS (HR, 0.22 [95% CI, 0.05–0.85], *p* = 0.029), and RFS (HR, 0.21 [95% CI, 0.05–0.90], *p* = 0.035).

### 3.5. Risk Stratification

**Figure 5:**
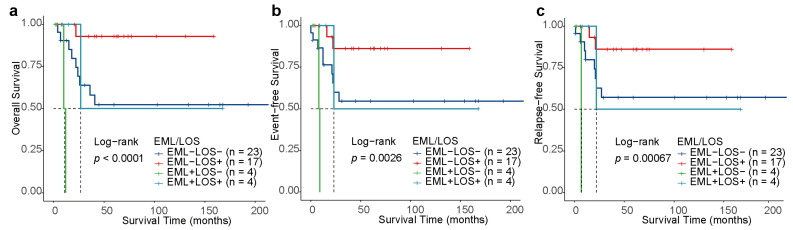
Kaplan–Meier curves for risk stratification. Kaplan–Meier analysis of OS (**a**), EFS (**b**) and RFS (**c**) for patients stratified by risk levels depending on EML and LOS.

To provide an estimation of prognostic stratification, we classified individuals into four risk groups based on EML and LOS: the EML+LOS+ (n = 4), EML+LOS− (n = 4), EML−LOS+ (n = 17), and EML−LOS− (n = 23) subgroups. As for other secondary ACAs that accompany t(8;21), one out of four individuals in the EML+LOS− subgroup had trisomy 4, 1 out of 23 individuals in the EML−LOS− group and 2 out of 17 patients in the EML−LOS+ subgroup had del(9q), and no patients had trisomy 8. Kaplan–Meier survival analysis unveiled that t(8;21) pAML patients with EML+LOS− had the most inferior outcome, while those with EML−LOS+ had the best prognosis, showing the following statistically significant survivals: the 3-year OS [0 vs. 92.9% (95% CI, 80.3–100), *p* < 0.001], the 3-year EFS [0 vs. 86.2% (95% CI, 70.0–100), *p* < 0.001], and the 3-year RFS [0 vs. 86.2% (95% CI, 80.3–100), *p* < 0.001] (Figure 5a–c). Given that the number of patients in the EML+LOS− and EML+LOS− subgroups was too small, we need to exercise caution when explaining the impact of risk stratification on prognosis. The observation of distinct prognostic effects between the two groups based on risk stratification suggested that EML+LOS− should be differentiated from EML−LOS+ in future research.

## 4. Discussion

This study demonstrated that, among a large cohort of 268 pAML individuals, 50 (18.7%) harbored t(8;21) translocation. This finding aligns with the range reported in other studies [29,30]. We explored the prognostic factors of t(8;21) pAML patients at a single Chinese children’s medical center. Previous studies demonstrated that t(8;21) pAML individuals likely benefit from regimens incorporating high doses of Ara-C during induction therapy [29]. In this study, the three-year OS, EFS, and RFS were 68.4% (95% CI, 55.0–85.1), 64.2% (95% CI, 50.7–81.4), and 65.5% (95% CI, 51.9–82.8), respectively, which were greater than that in the research by Wu et al. [31], comparable with the research by Che et al. [32], and obviously lower than that reported by a Japanese team [8]. Their better therapeutic effect may be attributed to a higher cumulative dose of Ara-C (59.4–78.4 g/m^2^) and adequate supportive care.

There was no difference in CR rate among different induction protocols [33,34]. In accordance with these studies, our cohort showed that patients treated with CHFU-AML 2005, CCLG-AML 2015, and CALSIII-AML18 regimens also showed no significant difference in CR rate (*p* > 0.05). Walter et al. [35] demonstrated that CR is a unique clinical significance factor in trials of de novo AML. Fang et al. [34] observed that AML individuals achieving CR1 showed better prognoses than those without CR1. In our study, however, we did not verify their finding that the clinical and biological features of individuals between CR1 and PR/NR groups were compared, but there was no statistical difference in CR rate among all factors (*p* > 0.05). Our results were slightly different from previous studies, considering that the sample size included in our study was small and needs to be further expanded in future studies.

In line with prior reports, the prevalence of ACAs was high (72.9%), and the most common cytogenetic aberration was LOS [36,37,38,39,40,41]. According to the Cox model, EML and LOS were independent prognostic factors for t(8;21) pAML individuals. Previous studies found that loss of Y chromosome (LOY) was linked to a high relapse risk and shorter OS [42,43]. However, some studies demonstrated that LOS had no significant associations with survival probabilities [19,36,44,45]. Mitterbauer et al. [46] suggested that LOS was linked to a significantly better EFS outcome. In our study, LOS was correlated with significantly better OS, EFS, and RFS, aligning with the research by Chen et al. [47].

The prognostic values of EML remain controversial across studies. Previous studies found that t(8;21) pAML patients with EML were linked to a low CR rate and poor RFS and OS [48,49,50]. However, Bisschop et al. [51] did not find significant associations with survival outcomes. The differences in results between studies may relate to the small-scale cohort, differences in race, and the wide range of case cohorts. In our study, EML was identified as an independent prognostic factor that is correlated with worse OS, EFS, and RFS, in line with the findings reported by Stove et al. [49] In addition, some reports claimed that patients with EML involving different sites had different survival outcomes [52,53]. In our study, subgroup analysis for the prognosis effect of EML at different sites could not be conducted due to the insufficient number of cases. Furthermore, individuals were categorized into four risk groups based on the two independent prognostic factors, LOS and EML, enabling the refinement of patients with different survival outcomes and suggesting that MRD should be closely monitored, as well as that HSCT should be considered in time among those subgroups with worse outcomes. To enhance the robustness of the findings, a larger cohort involving multiple medical centers is required for further verification of the results.

*C-KIT* mutations are reported to occur in 12−46% of adult t(8;21) AML patients [52,53,54,55], whereas they account for approximately 17−43% in pediatric t(8;21) patients [29,53,56,57,58,59]. The frequency of *C-KIT* in this study reached 60.0% (12/20), surpassing previous pediatric series [30,60]. This discrepancy could be owing to factors such as the small-sized sample from a single center, as well as the variations in the detection method and sequencing depth and coverage. Although *C-KIT* mutations mediate an adverse prognostic impact on the prognosis of adults with AML [61,62], the impact of *C-KIT* on pAML is still inconclusive [57,63]. Chen et al. [30] observed that *C-KIT* mutations are genetic markers linked to inferior prognoses in pAML patients. Another study indicated that *C-KIT* mutations were strongly correlated with poor outcome in t(8;21) pAML individuals [58]. In our study, despite the high frequency of *C-KIT* in t(8;21) pAML, it did not impact long-term prognosis, in line with previous pediatric reports [29,34]. This suggests potential differences in prognostic factors between pediatric and adult individuals diagnosed with this disease.

MRD is a pivotal marker in modern studies of pAML, with a commonly accepted threshold set at 0.1% [64]. MRD levels exceeding this threshold are linked to an elevated risk of relapse and unfavorable prognosis. In our study, MRD levels were quantified using MFCM following the first and second induction therapy phases in the CALSIII-AML18 protocol. However, the definitive prognostic value of MRD status derived from our findings may be inconclusive, likely due to the limited sample size.

The primary limitation of our study stems from its retrospective design, which may lead to missing data and an unavoidable bias. The power of the subsequent stratified analysis is constrained by the relatively small size of the subgroups and prospective research with a large-scale sample size is warranted to be practicable.

## 5. Conclusions

In conclusion, LOS is prognostically favorable, whereas EML is deemed to be strongly correlated with adverse prognoses in pediatric patients with t(8;21) AML. LOS combined with EML may help improve risk stratification and potentially facilitate the customization of future therapies.

## Figures and Tables

**Table 1:** Baseline features of 48 pediatric acute myeloid leukemia (pAML) patients with t(8;21).

Characteristics	n = 48
Gender, n (%)	
Male	31 (64.6)
Female	17 (35.4)
Age (M[range]) (years), n (%)	8.6 (2.5–16.7)
<3	1 (2.1)
3–14	45 (93.8)
>14	2 (4.2)
HB (M[range]) (g/L)	78.6 (38.2–122.0)
≤60	10 (20.8)
60–90	24 (50.0)
>90	14 (29.2)
PLT (M[range]) (×10^9^/L)	28.5 (4.0–265.0)
≤20	11(22.9)
20–100	34 (70.8)
>100	3 (6.3)
PB blast (M[range]) (%) ^1^, n (%)	45.0 (5.0–88.0)
BM blast (M[range]) (%), n (%)	51.25 (5.0–87.0)
FAB	
M2	33 (68.8)
M4	10 (20.8)
M5	3 (6.2)
NOS	2 (4.2)
Immunological classification, n (%)	
CD19 (+)	23 (47.9)
CD56 (+)	21 (43.6)
CD34 (+)	47 (97.9)
HLA-DR (+)	48 (100)
CD117 (+)	48 (100)
MPO (+)	34 (70.8)
CD13 (+)	20 (41.7)
CD33 (+)	35 (72.9)
Karyotype, n (%)	
CK	7 (14.6)
LOS	21 (43.8)
MK	11(22.9)
del(9q)	3 (6.3)
trisomy 4	1 (2.1)
Genetic mutations ^2^, N (%)	from 2018, N = 20
*C-KIT* (+)	12 (60.0)
*FLT3-ITD* (+)	2 (10.0)
Induction I MRD (%) ^3^, n (%)	from 2018, N = 20
<0.1	7 (35.0)
≥0.1	13 (65.0)
Induction II MRD (%) ^3^, n (%)	from 2018, N = 20
<0.1	18 (90.0)
≥0.1	2 (10.0)
EML ^4^, n (%)	8 (16.7)
CR1, n (%)	
CR	36 (75.0)
NR	5 (10.4)
PR	7 (14.6)
CR2, n (%)	
CR	46 (95.8)
NR	2 (4.2)
Year of diagnosis, n (%)	
2005–2014	18 (37.5)
2015–2018	10 (20.8)
2019–2023	20 (41.7)
HSCT, n (%)	9 (18.8)
Relapse, n (%)	13 (27.1)
Death, n (%)	13 (27.1)

Abbreviations: WBC: white blood cell counts; MRD: minimal residual disease; BM blast: bone marrow leukemic blast percentage; PLT: platelets; LOS: loss of sex chromosome; CR1: first complete remission; HB: hemoglobin; MK: monosomal karvotype; EML: extramedullary leukemia; PR: partial remission; NR: no remission; PB blast: peripheral blast percentage; del(9q), Deletion of chromosome 9q; M: median; CR2: second complete remission; CK: complex karyotype. ^1^ Missing data are due to being not submitted to the statistics center. N = 46. ^2^ Genetic mutation detection has been available since June 2018. N = 20. ^3^ MRD detection has been available since June 2018. N = 20. ^4^ EML was defined as the process whereby malignant leukemic cells from the BM infiltrate into tissues outside the BM at diagnosis, including the central nervous system, cutis, and periosteum, leading to the formation of an extramedullary mass.

**Table 2:** Univariate and multivariate logistic analyses of first complete remission (CR1) in 48 patients with t(8;21) AML.

Characteristics	CR	CRR	Univariate			Multivariate		
			OR	95% CI	*p* Value	OR	95% CI	*p* Value
Gender								
Female	11	64.7	Ref					
Male	25	80.6	2.27	0.59–8.89	0.228			
Age at diagnosis (years)								
≤8.6	21	84.0	Ref			Ref		
>8.6	15	65.2	0.36	0.08–1.35	0.141	0.55	0.08–3.25	0.514
WBC (×10^9^/L)								
>20	11	61.1	Ref			Ref		
≤20	25	83.3	3.18	0.84–12.99	0.093	1.86	0.34–10.32	0.464
PB blast (%)								
<45	20	83.3	Ref			Ref		
≥45	14	58.3	0.23	0.05–0.94	0.053	0.24	0.03–1.29	0.119
BM blast (%)								
<50	16	0.8	Ref					
≥50	20	71.4	0.62	0.15–2.37	0.501			
FAB								
M2	27	81.8	Ref			Ref		
M4	5	50.0	0.22	0.05–1.02	0.053	0.22	0.03–1.41	0.120
M5	2	66.7	0.44	0.04–10.47	0.534	0.90	0.03–36.68	0.988
NOS	2	100	3,478,080	0–NA	0.993	1,881,857	0-NA	0.996
CD19								
No	21	84.0	Ref			Ref		
Yes	15	65.2	0.36	0.08–1.35	0.141	0.66	0.04–7.82	0.752
CD56								
No	21	77.8	Ref					
Yes	15	71.4	0.71	0.19–2.70	0.615			
LOS								
No	21	77.8	Ref					
Yes	15	71.4	0.71	0.19–2.70	0.615			
CK								
No	29	70.7	Ref					
Yes	7	100	4,785,467	0-NA	0.996			
MK								
No	26	70.3	Ref			Ref		
Yes	10	90.9	4.23	0.68–82.44	0.193	3.62	0.36–87.81	0.318
EML								
No	29	72.5	Ref					
Yes	7	87.5	2.66	0.40–52.62	0.386			
Year of diagnosis								
2005–2014	16	88.9	Ref			Ref		
2015–2018	6	60.0	0.19	0.02–1.21	0.091	0.42	0.02–7.38	0.540
2019–2023	14	70.0	0.29	0.04–1.5	0.168	0.48	0.02–16.72	0.666

Abbreviations: WBC: white blood cell counts; LOS: loss of sex chromosome; HB: hemoglobin; MK: monosomal karvotype; PB_blast: peripheral blast percentage; EML: extramedullary leukemia; BM blast: bone marrow leukemic blast percentage; CRR: complete remission rate; PLT: platelets; CI: confidence interval; CK: complex karyotype; OR: odds ratio.

**Table 3:** Univariate and multivariate COX analyses of event-free survival (EFS) in 48 patients with t(8;21) AML.

Characteristics	Univariate			Multivariate		
	HR	95% CI	*p* Value	HR	95% CI	*p* Value
Gender						
Female	Ref					
Male	1.63	0.51–5.20	0.412			
Age at diagnosis (years)						
≤8.6	Ref					
>8.6	1.55	0.54–4.42	0.417			
WBC (×10^9^/L)						
>20	Ref					
≤20	0.76	0.25–2.28	0.623			
PB blast (%)						
<45	Ref					
≥45	1.48	0.47–4.67	0.502			
BM blast (%)						
<50	Ref					
≥50	1.10	0.38–3.17	0.864			
FAB						
M2	Ref					
M4	0.29	0.04–2.25	0.236			
M5	0.98	0.13–7.61	0.984			
NOS	1.83	0.24–14.21	0.564			
CD19						
No	Ref					
Yes	0.53	0.17–1.68	0.28			
CD56						
No	Ref					
Yes	0.65	0.22–1.95	0.443			
LOS						
No	Ref			Ref		
Yes	0.26	0.07–0.92	0.036	0.22	0.05–0.85	0.029
CK						
No	Ref					
Yes	1.12	0.25–5.04	0.878			
MK						
No	Ref					
Yes	0.57	0.13–2.56	0.464			
*C-KIT*						
No	Ref					
Yes	1.99	0.2–19.39	0.554			
*FLT3-ITD*						
No	Ref					
Yes	NA	NA	NA			
Induction I MRD (%)						
<0.1	Ref					
≥0.1	0	0-Inf	0.999			
Induction II MRD (%)						
<0.1	Ref					
≥0.1	4.29	0.38–48.77	0.241			
EML						
No	Ref			Ref		
Yes	2.52	0.69–9.24	0.164	4.75	1.10–20.61	0.037
CR1						
CR	Ref					
not in CR	0.91	0.25–3.27	0.885			
CR2						
CR	Ref					
not in CR	∞	0-Inf	1.00			
Year of diagnosis						
2005–2014	Ref					
2015–2018	1.21	0.35–4.13	0.766			
2019–2023	0.64	0.16–2.5	0.523			

Abbreviations: WBC: white blood cell counts; MK: monosomal karvotype; BM blast: bone marrow leukemic blast percentage; CK: complex karyotype; PLT: platelets; LOS: loss of sex chromosome; HB: hemoglobin; CR1: first complete remission; PB blast: peripheral blast percentage; EML: extramedullary leukemia; HR: hazard ratio; CR2: second complete remission; CI: confidence interval.

## Data Availability

Data are provided within the manuscript or Supplementary Information files.

## Supplementary Materials

The following supporting information can be downloaded at: https://www.mdpi.com/article/10.3390/children11050605/s1. Figure S1. Probability of survival for patients with t(8;21) AML in the protocols. Figure S2. Probability of survival depending on the *C-KIT* mutation in t(8;21) AML. Kaplan–Meier survival curves of OS (a), EFS (b) and RFS (c) for patients stratified by *C-KIT* mutation. Figure S3. Probability of survival depending on the Induction I MRD status in t(8;21) AML. Kaplan–Meier survival curves of OS (a), EFS (b) and RFS (c) for patients stratified by Induction I MRD status. Figure S4. Probability of survival depending on the Induction II MRD status in t(8;21) AML. Kaplan–Meier survival curves of OS (a), EFS (b) and RFS (c) for patients stratified by Induction II MRD status. Table S1. Definitions of end points. Table S2. Comparison of characteristics for patients with t(8;21) AML in differrent consecutive protocols. Table S3. Univariate and multivariate COX analyses of OS in 48 patients with t(8;21) AML. Table S4. Univariate and multivariate COX analyses of RFS in 48 patients with t(8;21) AML. Table S5. Univariate and multivariate COX analyses of overall survival (OS) in 48 patients with t(8;21) AML. Table S6. Univariate and multivariate COX analyses of overall survival (OS) in 20 t(8;21) AML patients who enrolled after 2018. Table S7. Univariate and multivariate COX analyses of relapse-free survival (RFS) in 48 patients with t(8;21) AML. Table S8. Univariate and multivariate COX analyses of relapse-free survival (RFS) in 20 t(8;21) AML patients who enrolled after 2018.
