# Supplementary material for: Prognostic Factors of Pediatric Acute Myeloid Leukemia Patients with t(8;21) (q22;q22): A Single-Center Retrospective Study
Source: Children (Basel). 2024 May 17;11(5):605. doi: 10.3390/children11050605 (PMC11120327; doi:10.3390/children11050605)
Supplement: Supplementary file 1 [file children-11-00605-s001.zip › children-2997202-supplementary.pdf]

Supplementary Information

Prognostic Factors of Pediatric Acute Myeloid Leukemia Patients with t(8;21) (q22;q22): A Single-Center Retrospective Study

Jiapeng Yang, Xiaohua Zhu, Honghong Zhang, Yang Fu, Zifeng Li, Ziping Xing, Yi Yu, Ping Cao, Jun Le, Junye Jiang, Jun Li, Hongsheng Wang and Xiaowen Zhai *

Department of Hematology and Oncology, National Children's Medical Center, Children's Hospital of Fudan University, Shanghai, China

* Correspondence: Xiaowen Zhai, Department of Hematology and Oncology, National Children's Medical Center, Children's Hospital of Fudan University, Shanghai, 201102, China. Email: xwzhai@fudan.edu.cn.

Table S1. Definitions of end points.

End point	Abbreviation	Definition
Complete remission	CR	Bone marrow shows normal hematopoiesis, bone marrow blasts less than 5%, no blasts with Auer rods or persistence of extramedullary leukemia, regeneration of normal cell production in peripheral blood (absolute neutrophil count > 1×10^9 /L, platelets ≥ 100×10^9 /L).
Partial remission	PR	Bone marrow blasts 6-19%.
No remission	NR	Bone marrow blasts more than 20%.
Event-free survival	EFS	The time from the date of diagnosis to the date of first event (relapse, death of any cause, failure to achieve remission, or secondary malignancy) or last follow-up. Failure to attain morphological remission was considered an event on day 0.
Relapse-free survival	RFS	The time from the date of achieving CR1 until the first relapse, death or the final follow-up day.
Overall survival	OS	The time from the date of diagnosis to the date of last follow-up or death.
Event		
Relapse		Reappearance of leukemic blasts in the peripheral blood, or the finding of more than 5% blasts in the bone marrow, or extramedullary relapse.
Secondary malignancy		
Death		

Abbreviations: CR1, first complete remission.

Table S2. Comparison of characteristics for patients with t(8;21) AML in differrent consecutive protocols.

Protocol	CHFU-AML 2005	CCLG-AML-2015	CALSIII-AML18	P Value
Period	2005-2014	2015-2018	2019-2023	
Number of patients	18	10	20	
age (M[range]) (months)	96.0 (30.0, 168.0)	140.5 (39.0, 164.0)	102.5 (38.0, 200.0)	0.388
WBC (M[range]) ($\times 10^9$ /L)	16.2 (1.7, 120.6)	18.1 (3.7, 123.0)	15.5 (5.7, 47.0)	0.996
PB blast (M[range])(%)	48.0 (10.0, 88.0)	44.0 (8.0, 70.0)	45.0 (0.0, 82.0)	0.787
BM blast (M[range]) (%)	50.0 (21.5, 87.0)	44.1 (16.5, 80.5)	55.5 (5.0, 76.0)	0.648
PLT (M[range]) ($\times 10^9$ /L)	25.5 (4.0, 154.0)	33.5 (15.0, 96.0)	24.5 (11.0, 265.0)	0.320
HB (M[range]) (g/L)	80.1 (38.2, 122.0)	75.6 (44.0, 122.0)	74.5 (39.0, 120.0)	0.932
Gender				0.253
Female	9 (50.0)	3 (30.0)	5 (25.0)	
Male	9 (50.0)	7 (70.0)	15 (75.0)	
FAB				0.232
M2	13 (72.2)	4 (40.0)	16 (80.0)	
M4	2 (11.1)	4 (40.0)	4 (20.0)	
M5	2 (11.1)	1 (10.0)	0 (0.0)	
NOS	1 (5.6)	1 (10.0)	0 (0.0)	
LOS				0.217
No	13 (72.2)	5 (50.0)	9 (45.0)	
Yes	5 (27.8)	5 (50.0)	11 (55.0)	
MK				0.950
No	14 (77.8)	8 (80.0)	15 (75.0)	
Yes	4 (22.2)	2 (20.0)	5 (25.0)	
CK				
No	17 (94.4)	7 (70.0)	17 (85.0)	0.213
Yes	1 (5.6)	3 (30.0)	3 (15.0)	
EML				0.423
No	16 (88.9)	9 (90.0)	15 (75.0)	
Yes	2 (11.1)	1 (10.0)	5 (25.0)	
CR1				0.190
CR	16 (88.9)	6 (60.0)	14 (70.0)	
not in CR	2 (11.1)	4 (40.0)	6 (30.0)	
CR2				0.405
CR	17 (94.4)	9 (90.0)	20 (100)	

not in CR	1 (5.6)	1 (10.0)	0 (0.0)	
Survival status				0.262
Censored	12 (66.7)	6 (60.0)	17 (85.0)	
Death	6 (33.3)	4 (40.0)	3 (15.0)	

Abbreviations: WBC: White blood cell counts; HB: Hemoglobin; PLT: Platelets; PB blast: Peripheral blast percentage; BM blast: Bone marrow leukemic blast percentage; FAB: French-American-British classification; CR1: First complete remission; CR2: Second complete remission; LOS : Loss of sex chromosome; MK: Monosomal karvotype; EML: Extramedullary leukemia; M: median; CK: Complex karyotype.

Table S3. Univariate and multivariate logistic analyses of first complete remission in 20 t(8;21) AML patients who enrolled after 2018.

Characteristics	CR	CRR	Univariate			Multivariate		
			OR	95%CI	P Value	OR	95%CI	P Value
**C-KIT**								
No	6	66.7	Ref					
Yes	8	66.7	1.00	0.15-6.35	1.000			
**FLT3/ITD**								
No	13	50.0	Ref					
Yes	1	68.4	0.46	0.02-13.01	0.606			

Abbreviations: CR: Complete remission; CRR: Complete remission rate; OR: Odds ratio; CI: Confidence interval; CK: Complex karyotype.

Table S4. Univariate and multivariate COX analyses of event-free survival (EFS) in 20 t(8;21) AML patients who enrolled after 2018.

Characteristics	Univariate			Multivariate		
	HR	95%CI	P Value	HR	95%CI	P Value
**C-KIT**						
No	Ref					
Yes	1.99	0.2-19.39	0.554			
**FLT3/ITD**						
No	Ref					
Yes	NA	NA	NA			
**Induction I MRD (%)**						
<0.1	Ref					
≥0.1	0	0-Inf	0.999			
**Induction II MRD (%)**						
<0.1	Ref					
≥0.1	4.29	0.38-48.77	0.241			

Abbreviations: WBC: White blood cell counts; HB: Hemoglobin; PLT: Platelets; PB blast: Peripheral blast percentage; BM blast: Bone marrow leukemic blast percentage; FAB: French-American-British classification; LOS: Loss of sex chromosome; MK: Monosomal karvotype; CK: Complex karyotype; CR1: First complete remission; CR2: Second complete remission; EML: Extramedullary leukemia; HR: hazard ratio; CI: confidence interval.

Table S5. Univariate and multivariate COX analyses of overall survival (OS) in 48 patients with t(8;21) AML.

Characteristics	Univariate			Multivariate		
	HR	95%CI	P Value	HR	95%CI	P Value
Gender						
Female	Ref					
Male	1.53	0.47-4.97	0.483			
Age at diagnosis (years)						
≤8.6	Ref					
>8.6	1.33	0.45-3.99	0.605			
WBC (×10^9/L)						
>20	Ref					
≤20	0.68	0.22-2.08	0.499			
PB blast (%)						
<45	Ref					
≥45	1.20	0.37-3.93	0.765			
BM blast (%)						
<45	Ref					
≥45	1.01	0.34-3.02	0.981			
FAB						
M2	Ref					
M4	0.33	0.04-2.55	0.285			
M5	1.01	0.13-7.94	0.990			
NOS	2.03	0.26-15.93	0.500			
CD19						
No	Ref					
Yes	0.61	0.19-1.99	0.416			
CD56						
No	Ref					
Yes	0.76	0.25-2.32	0.629			
LOS						
No	Ref			Ref		
Yes	0.17	0.04-0.77	0.022	0.08	0.01-0.48	0.005
CK						
No	Ref					
Yes	1.36	0.30-6.15	0.688			
MK						
No	Ref					
Yes	0.69	0.15-3.14	0.635			
EML						
No	Ref			Ref		

Yes	3.35	0.9-12.42	0.071	10.99	2.08-58.12	0.005
CR1						
CR	Ref					
not in CR	0.99	0.27-3.61	0.990			
CR2						
CR	Ref					
not in CR	∞	0-Inf	1.000			
Year of diagnosis						
2005-2014	Ref					
2015-2018	1.33	0.38-4.72	0.658			
2019-2023	0.88	0.22-3.57	0.864			

Abbreviations: WBC: White blood cell counts; HB: Hemoglobin; PLT: Platelets; PB blast: Peripheral blast percentage; BM blast: Bone marrow leukemic blast percentage; FAB: French-American-British classification; LOS : Loss of sex chromosome; MK: Monosomal karyotype; CR1: First complete remission; CR2: Second complete remission; EML: Extramedullary leukemia; HR: hazard ratio; CI: confidence interval; CK: Complex karyotype.

Table S6. Univariate and multivariate COX analyses of overall survival (OS) in 20 t(8;21) AML patients who enrolled after 2018.

Characteristics	Univariate			Multivariate		
	HR	95%CI	P Value	HR	95%CI	P Value
C-KIT						
No	Ref					
Yes	1.48	0.15-14.39	0.736			
FLT3/ITD						
No	Ref					
Yes	NA	NA	NA			
Induction I MRD (%)						
<0.1	Ref					
⩾0.1	0	0-Inf	0.999			
Induction II MRD (%)						
<0.1	Ref					
⩾0.1	3.72	0.33-41.46	0.286			

Abbreviations: HR: hazard ratio; CI: confidence interval; MRD: Minimal residual disease.

Table S7. Univariate and multivariate COX analyses of relapse-free survival (RFS) in 48 patients with t(8;21) AML.

Characteristics		Univariate			Multivariate	
	HR	95%CI	P Value	HR	95%CI	P Value
Gender						
Female	Ref					
Male	2.20	0.60-8.01	0.232			
Age at diagnosis (months)						
≤8.6	Ref					
>8.6	1.36	0.46-4.06	0.580			
WBC (×10^9/L)						
>20	Ref					
≤20	0.92	0.28-2.99	0.887			
PB blast (%)						
<45	Ref					
≥45	1.27	0.39-4.16	0.692			
BM blast (%)						
<50	Ref					
≥50	0.96	0.32-2.87	0.945			
FAB						
M2	Ref					
M4	0.29	0.04-2.27	0.240			
M5	0	0-Inf	0.998			
NOS	1.86	0.24-14.45	0.553			
CD19						
No	Ref					
Yes	0.60	0.19-1.96	0.399			
CD56						
No	Ref					
Yes	0.73	0.24-2.23	0.577			
LOS						
No	Ref			Ref		
Yes	0.28	0.08-1.00	0.051	0.21	0.05 - 0.90	0.035
CK						
No	Ref					
Yes	1.24	0.27-5.62	0.778			
MK						
No	Ref					
Yes	0.62	0.14-2.83	0.541			
EML						
No	Ref			Ref		

Yes	2.94	0.79-10.95	0.108	6.55	1.40-30.63	0.017
CR1						
CR	Ref					
not in CR	0.62	0.14-2.80	0.534			
CR2						
CR	Ref					
not in CR	∞	0-Inf	1.000			
Year of diagnosis						
2005-2014	Ref					
2015-2018	1.44	0.41-5.11	0.574			
2019-2023	0.79	0.20-3.19	0.745			

Abbreviations: WBC: White blood cell counts; HB: Hemoglobin; PLT: Platelets; PB blast: Peripheral blast percentage; BM blast: Bone marrow leukemic blast percentage; FAB: French-American-British classification; LOS : Loss of sex chromosome; MK: Monosomal karyotype; CR1: First complete remission; CR2: Second complete remission; EML: Extramedullary leukemia; HR: hazard ratio; CI: confidence interval; CK: Complex karyotype.

Table S8. Univariate and multivariate COX analyses of relapse-free survival (RFS) in 20 t(8;21) AML patients who enrolled after 2018.

Characteristics	Univariate			Multivariate		
	HR	95%CI	P Value	HR	95%CI	P Value
KIT						
No	Ref					
Yes	1.99	0.20-19.39	0.554			
FLT3/ITD						
No	Ref					
Yes	NA	NA	NA			
Induction I MRD (%)						
<0.1	Ref					
⩾0.1	0	0-Inf	0.999			
Induction II MRD (%)						
<0.1	Ref					
⩾0.1	4.29	0.38-48.77	0.241			

Abbreviations: HR: hazard ratio; CI: confidence interval; CK: Complex karyotype; MRD: Minimal residual disease.

Supplementary Figure

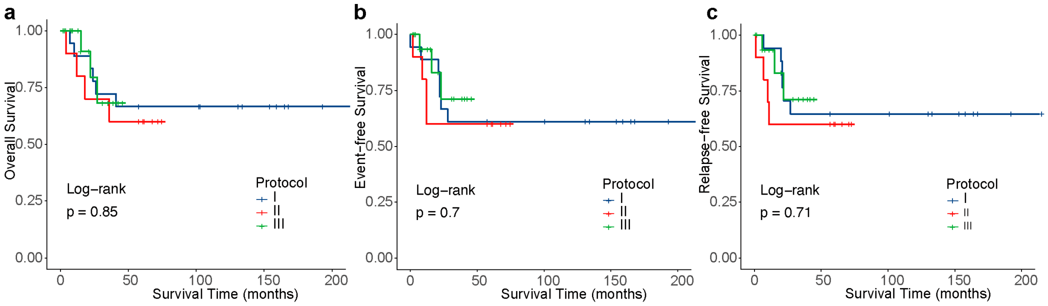

Figure S1. Probability of survival for patients with t(8; 21) AML in the protocols: (I) CHFU-AML 2005, (II) CCLG-AML 2015, (III) CALSⅢ-AML18. (a) Probabilities of 3-year overall survival (OS) ± standard deviations (SD): 0.72 ± 0.11, 0.60 ± 0.16, 0.68 ± 0.15 in periods I–III, respectively. (b) Probabilities of three-year event-free survival (EFS) ± SD: 0.62 ± 0.12, 0.60 ± 0.16, 0.71 ± 0.15 in periods I–III, respectively. (c) Probabilities of three-year relapse-free survival (RFS) ± SD: 0.65 ± 0.12, 0.60 ± 0.16, 0.71 ± 0.15 in periods I–III, respectively.

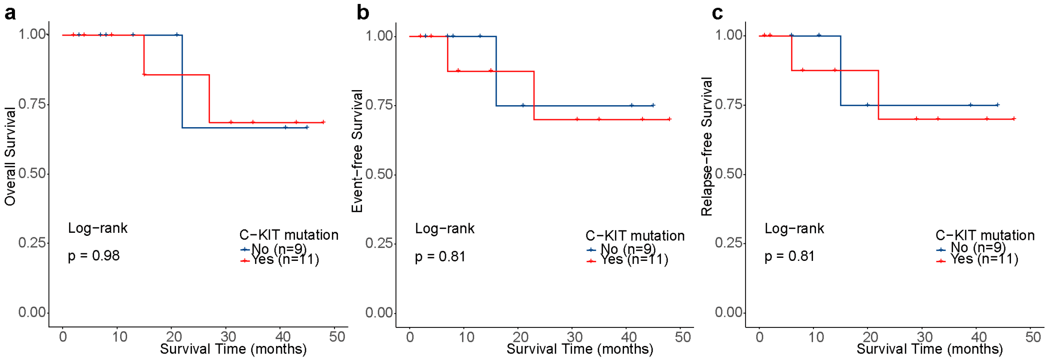

Figure S2. Probability of survival depending on the C-KIT mutation in t(8;21) AML. Kaplan–Meier survival curves of overall survival (OS) (**a**), event-free survival (EFS) (**b**) and relapse-free survival (RFS) (**c**) for patients stratified by C-KIT mutation.

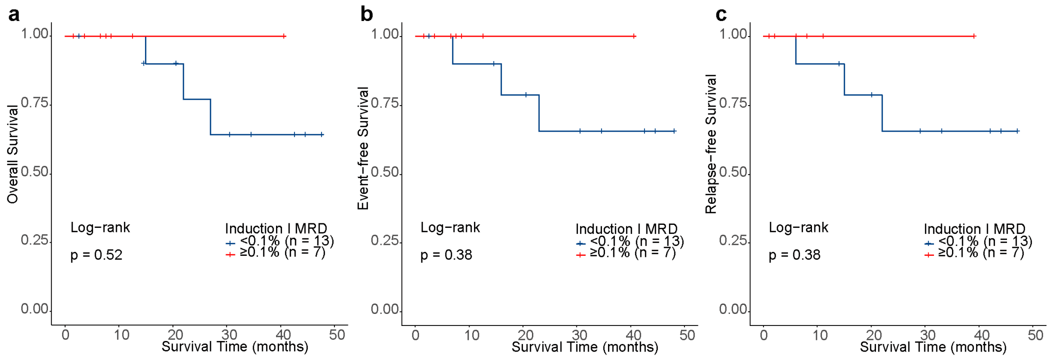

Figure S3. Probability of survival depending on the Induction I Minimal residual disease (MRD) status in t(8;21) AML. Kaplan–Meier survival curves of overall survival (OS) (**a**), event-free survival (EFS) (**b**) and relapse-free survival (RFS) (**c**) for patients stratified by Induction I MRD status.

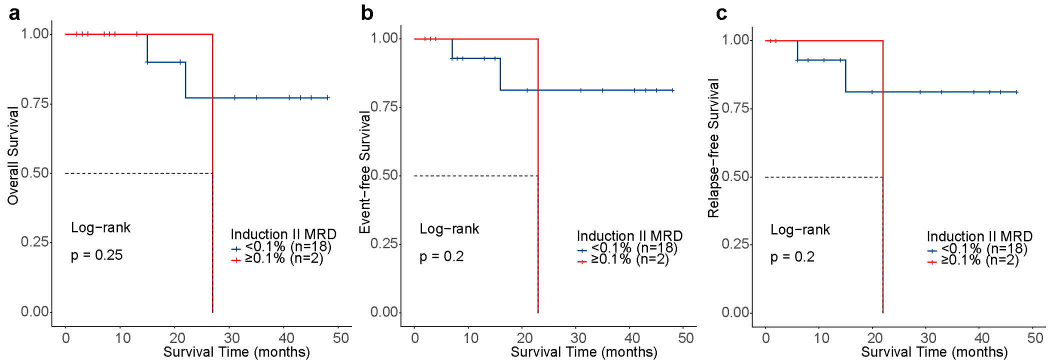

Figure S4. Probability of survival depending on the Induction II Minimal residual disease (MRD) status in t(8;21) AML. Kaplan–Meier survival curves of overall survival (OS) (**a**), event-free survival (EFS) (**b**) and relapse-free survival (RFS) (**c**) for patients stratified by Induction II MRD status.
